# Association between ablation-induced baroreceptor reflex modification and procedure efficacy in patients with atrial fibrillation

**DOI:** 10.3389/fcvm.2024.1474002

**Published:** 2024-10-14

**Authors:** Anna Zuk, Roman Piotrowski, Agnieszka Sikorska, Ilona Kowalik, Piotr Kulakowski, Jakub Baran

**Affiliations:** ^1^ Department of Cardiology, Centre of Postgraduate Medical Education, Grochowski Hospital, Warsaw, Poland; ^2^ Clinical Research Support Center, National Institute of Cardiology, Warsaw, Poland

**Keywords:** baroreceptor reflex, atrial fibrillation, atrial fibrillation symptoms, ablation efficacy, autonomic nervous system

## Abstract

**Background:**

The autonomic nervous system (ANS) plays a significant role in atrial fibrillation (AF). Catheter ablation (CA) is a well-established treatment method for AF and significantly affects the ANS, including baroreceptor (BR) function. However, little is known about the changes in BR function caused by radiofrequency (RF) or cryoballoon energy (CB) and its impact on future AF recurrences.

**Purpose:**

To assess 1-year efficacy of CA of AF in relation to BR function modification and type of ablation energy used.

**Methods:**

The study group consisted of 78 patients (25 females, mean age 58 ± 9 years) with paroxysmal AF and first CA (39 patients in the RF group and 39 in the CB group). The BR function was assessed non-invasively, using tilt testing before and after CA, and three BR parameters were calculated: event count (BREC) depicting overall BR activity, slope mean depicting BR sensitivity (BRS), and BR effectiveness index (BEI). The efficacy of CA was assessed during 1-year follow-up, which consisted of ambulatory visits and 24-h Holter ECG recordings at 3, 6, and 12 months after CA. The quality of life was assessed by using a dedicated scale [University of Toronto Atrial Fibrillation Severity Scale (AFSS)].

**Results:**

The two groups did not show differences in terms of clinical or demographic data. One-year follow-up was completed for 35 (89.7%) patients from the CB group and for 34 (87.2%) from the RF group. The rates of efficacy of CB and RF were similar [31/35 (88.6%) vs. 26/34 (76.5%), respectively]. After CA, the BR function decreased in both groups, with a significantly greater decrease in the CB group. The changes in BR parameters were similar in both responders and non-responders after CA in the whole group [BREC 10.0 (2.0–24.0) vs. 12.0 (4.0–21.5), *p* = 0.939; BRS 5.4 (3.7–6.5) vs. 4.8 (3.6–7.2), *p* = 0.809; BEI 24.8 (15.9–27.4) vs. 17.5 (8.9–27.5), *p* = 0.508, respectively]. According to the AFSS, the AF symptoms were significantly reduced in both groups to a similar extent.

**Conclusions:**

CA for AF significantly decreased the BR function, especially in patients undergoing CB. There was no correlation between CA-induced changes in BR parameters and ablation outcome.

## Background

Atrial fibrillation (AF) affects 2%–4% of the adult population (1) and is linked to higher morbidity, including heart failure and ischemic stroke (2). The autonomic nervous system (ANS) plays a significant role in the etiology of AF (3). Catheter ablation (CA) is a well-established treatment method for AF, which is performed using radiofrequency (RF) current or freezing with cryoballoon (CB), but it significantly affects the ANS, including baroreceptor (BR) function (4, 5). However, little is known about the changes in BR function caused by RF or CB energy and their relationship with the long-term efficacy of AF ablation. During the ablation procedure, not only are the pulmonary veins (PVs) isolated but also a modification of the cardiac ganglionated plexi (GP) located in the epicardial pad is performed. Some studies reported that the modification of the ANS improved the efficacy of PV isolation (PVI) and prevented AF recurrences (4, 6).

The effects of PVI on the ANS have been investigated in several studies, mainly by assessing heart rate (HR) and heart rate variability (HRV) (7, 8). However, these parameters have several limitations when assessing ANS function, whereas examining BR function may offer a more accurate ANS assessment. We previously showed that CA for AF significantly decreased the BR function and that this decrease was more pronounced following CB than RF CA (9). However, the relationship between CA-induced changes in BR function and efficacy of CA as well as quality of life (QoL) has not been examined yet.

### Aims

To assess 1-year efficacy of CA of AF in relation to BR function modification and type of ablation energy used.

## Methods

### Study group

In the period between 2016 and 2018, we performed an observational, prospective, single-center study (ClinicalTrials.gov Identifier: NCT03811639) that enrolled patients admitted for CA of paroxysmal AF—named ABLANSAF study (Changes in Cardiac Autonomic Nervous System Following Atrial Fibrillation Ablation). The study was approved by the Local Ethics Committee (No 65/PB/2015) and all participants provided written informed consent.

A total of 78 patients were included in the ABLANSAF study during this period and 69 patients completed a 1-year follow-up. The inclusion criteria were as follows: (1) paroxysmal AF, (2) first CA, (3) sinus rhythm at the time of BR measurements, (4) no sick sinus syndrome, (5) no implanted pacemaker or defibrillator, (6) no PV anatomy favoring any specific ablation technique (for example, the common trunk of the left PV favoring RF), (7) no additional cavo-tricuspid CA or another atrial linear application, and (8) no evidence of severe heart failure defined as left ventricular ejection fraction <35%.

### Study outline

**Figure 1:**
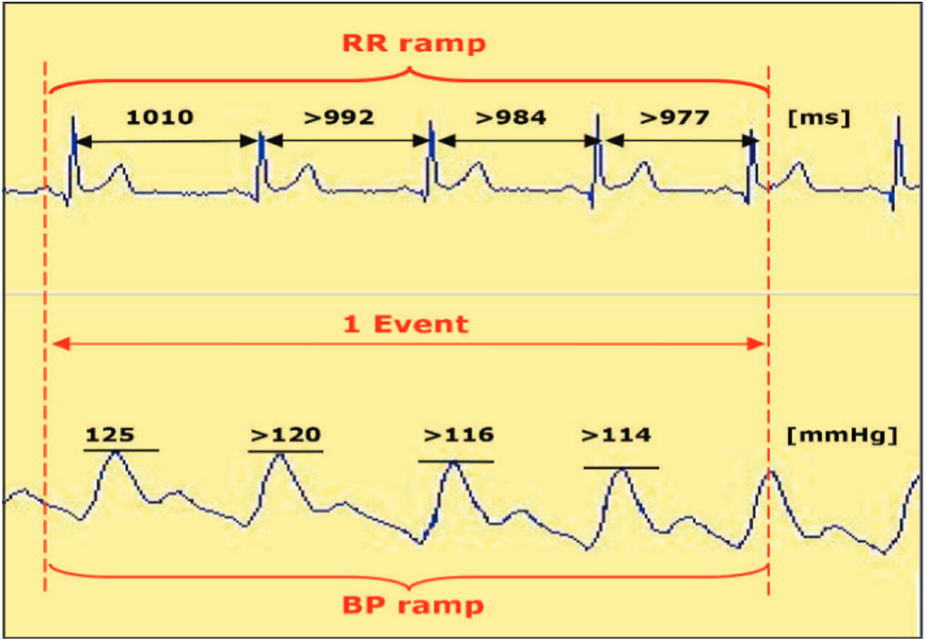
An example of spontaneous baroreflex sensitivity calculated using the so-called sequence method.

Patients were admitted to the hospital one day before the procedure. We assessed the BR function twice—before CA at 7:00 am on the day of the procedure and 2 days after CA. The BR parameters were measured during a shortened protocol of 25-min tilt test at rest, while supine, and after tilting (70°, no nitroglycerine challenge). The Task Force Monitor (TFM) software (CNSystem, 2007, version 2.2, Austria) was used for offline analysis (10). The producer of the Task Force Monitor CNS System declared under sole responsibility that the products were in compliance with directive 93/42/EEC. The TFM passed the tests for the CE quality mark (CE 0408, TUeV Austria, Vienna). TFM, certified in Europe, provides correct and reliable hemodynamic data (11). By identifying the sequences of at least three consecutive heartbeats with either a progressive increase in systolic blood pressure (SBP) and consequent prolongation of the R-R interval or a progressive decrease in SBP and resultant shortening of the R-R interval, the BR function was measured. The SBP and R-R interval changes must have been at least 1 mmHg over 4 ms. To determine BR sensitivity (BRS, in ms/mmHg), we set the interval between R-R and SBP values at 0 beats and computed the slope of the regression line between the R-R intervals and the SBP values for each sequence. An original example of ECG tracings with R-R intervals and SBP values (plethysmographic method) is shown in Figure 1.

Three BR parameters were calculated non-invasively during supine and tilt positions upon tilt testing. The following three parameters were assessed:

1. BR event count (BREC) depicting overall BR activity (the number of events per minute),
2. BR slope mean depicting BRS measured by the slope of the linear regression between the R-R intervals and the systolic BP values,
3. BR effectiveness index (BEI) that describes the effectiveness of baroreceptors and is measured by the proportion of events that take place divided by the total number of blood pressure (BP) changes.

Medications, such as beta blockers or antiarrhythmic drugs, remained unchanged before or directly after CA.

### Ablation procedures

Procedural details were previously described (9). The RF CA procedure was performed using a single transseptal puncture and a Thermocool SmartTouch catheter (Biosense Webster, USA). The CARTO 3 system, ablation index (550 AI on the anterior wall was 550, and 380 on the posterior wall) for the module, and the CLOSE protocol were used (12). Intracardiac echocardiography (ICE) was used during the performance of all procedures. PVI was confirmed using a diagnostic circular catheter or by pacing from inside the ablation line. CB was performed using a flexible 15F sheath (Flex-Cath Advance, Medtronic, USA) and second-generation CB (Arctic front 2AF281 balloon, Medtronic, USA) with a diagnostic Achieve electrode (Medtronic, USA) to confirm PVI.

### Follow-up

Patients were followed up for 1 year after CA. They underwent serial 4- to 7-day Holter ECG recordings (DMS 300-4A, DM Software, Stateline, NV, United States) at 3, 6, and 12 months after CA. Any antiarrhythmic medication was left unchanged up to 3 months after ablation and was stopped if no AF recurrence was observed. Effective ablation was defined as no AF, or atrial tachycardia (AT) episodes lasting longer than 30 s were recorded during Holter ECG or using standard ECG. The QoL was assessed using a dedicated scale (the University of Toronto Atrial Fibrillation Severity Scale—AFSS). The AFSS is a 19-item self-administered questionnaire designed to capture AF-related burden and its symptoms (frequency, duration, and severity of episodes) and healthcare utilization (13). The score of the scale is a summary that averages the frequency, duration, and patient-detected severity of AF episodes (14). The higher the score the greater the AF burden (13). We analyzed global wellbeing, the question about AF severity: higher score = more severe AF and the global number of points that characterize AF burden.

### Statistical analysis

Statistical analyses were conducted using SAS statistical package ver. 9.4 (SAS Institute Inc., Cary, NC, USA). Continuous variables are expressed as mean ± SD (normal distribution) or median and the 25th–75th percentile range for skewed distribution. Normality was verified using the Shapiro–Wilk test. Between-group comparisons were made using Student's *t*-test or the non-parametric Mann–Whitney test, as appropriate. Within-subject comparisons at two time points were performed using a paired Student's *t*-test or the non-parametric signed-rank test. Due to the skewness of the distributions, the correction in relation to the baseline measurements was carried out using the robust regression method. The strength of linear relationships between numerical variables was measured by using Spearman's correlation coefficient. The statistical significance of the coefficients was tested. Categorical data are reported as counts and percentages. The differences in proportion between groups were analyzed using Fisher's exact test or Pearson’s χ^2^ test. All statistical tests were treated as two-sided and evaluated at a significance level of 0.05.

## Results

Of the 78 patients (age 58 ± 9 years, 25 females), RF CA was performed in 39 patients and CB in 39 patients. The two groups of patients did not differ in terms of demographic and clinical characteristics (Table 1). The patients were on the following medications: B-blockers, propafenone, sotalol, verapamil, and amiodarone. In these groups, there were no significant differences between CB and RF (*p* = 0.80, *p* = 0.26, *p* = 1.00, *p* = 1.00, *p* = 0.71, respectively).

**Table 1:** A comparison of the baseline study population.

Parameter	Cryoballoon, *n* = 39	RF ablation, *n* = 39	*P*-value
Age (years)	57.5 ± 8.9	58.3 ± 9.9	0.719
Weight (kg)	87.6 ± 13.9	84.3 ± 11.2	0.251
BMI	28.3 ± 3.9	28.2 ± 3.0	0.871
Men	29 (74.4%)	24 (61.5%)	0.225
Heart failure	1 (2.6%)	3 (7.7%)	0.615
Hypertension	20 (51.3%)	28 (71.8%)	0.063
Diabetes	2 (5.1%)	5 (12.8%)	0.431
Stroke	3 (7.7%)	0 (0%)	0.240
Vascular disease	4 (10.3%)	4 (10.3%)	1.00
CHADS2VASc	1 [0; 2]	1 [0; 3]	0.217
Hyperlipidemia	16 (41.0%)	19 (48.7%)	0.495
Kidney disease	0 (0%)	4 (10.3%)	0.115
LA diameter	37.7 ± 4.0	38.3 ± 4.4	0.511
LVEF	60.4 ± 4.7	58.8 ± 6.3	0.198
HASBLED			
0	20 (51.3%)	19 (48.7%)	0.950
1	13 (33.3%)	13 (33.3%)	
2	6 (15.4%)	7 (18.0%)	
Medication			
Beta blockers	26/39 (67%)	28/39 (71%)	0.81
Propafenone	15/39 (38%)	21/39 (54%)	0.26
Sotalol	4/39 (10%)	4/39 (10%)	1.00
Amiodarone	3/39 (8%)	5/39 (13%)	0.71

BMI, body mass index; CHADS2VASc—congestive heart failure, hypertension, Age ≥75 years old diabetes, stroke, vascular disease, age 65–74, sex category; HASBLED—hypertension, abnormal liver/renal function, stroke, bleeding history or predisposition, labile INR, elderly; >65 years old, drugs/alcohol; LA, left atrium; LVEF, left ventricle ejection fraction.

### Baroreceptor function

As described previously (9), the baseline BR parameters, both while supine and after tilting, were similar in both groups. CA reduced all of the evaluated BR parameters in the whole group while supine and after tilting (9). The decrease in BRS parameters was more marked in the CB group than in the RF group when assessing BREC (9).

### CA efficacy

The 1-year follow-up was completed by 35 (89.7%) patients from the CB group and by 34 (87.2%) subjects from the RF group. After CA, all of the evaluated BR parameters decreased in both groups (9). A significant negative correlation was found between the difference in baroreceptor parameters (before and after ablation) and baseline values of baroreceptor parameters in both groups in the supine phase and after tilting. Regression coefficients showed significantly greater dependence of the decrease in BRS event count from the baseline values in CB vs. RF ablation. This relationship is statistically significant both for supine and tilt positions in the CB and RF groups [regression coefficient supine: −0.88 ±  0.11 vs. −0.45 ± 0.09; *p* = 0.003; tilt position: −0.99 ± 0.11 and −0.67 ± 0.07; *p* = 0.015, respectively). On repeated Holter ECG monitoring, the rate of efficacy of CB and RF was similar at 12 months (31/35 (88.6%) vs. 26/34 (76.5%); *p* = 0.185, respectively]. After CA, the BR function decreased in the whole group, but the values of the BR parameters did not differ between responders and non-responders, both while supine and after tilting (Table 2). The reduction in the BR values was not related to the efficacy depicted in Holter monitoring. The CA-induced changes (delta) in all BR parameters were similar in responders vs. non-responders, in both supine and tilt positions (Table 3). Although no significant differences were observed between responders and non-responders, in the RF ablation group, the BREC and BEI values in the supine position were approximately twofold lower in the supine position before and after ablation in responders compared with non-responders, which resulted in differences in BREC (after tilting, *p* = 0.047) and BEI (supine, *p* = 0.037) in responders (Table 4). There were no significant differences in BR parameters in non-responders and responders after ablation compared with baseline measurements in the supine and tilt positions in both groups (Table 5). However, data related to the above are underpowered because of the relatively small number of patients in both groups.

**Table 2:** Baroreceptor parameters in non-responders and responders before and after the ablation procedure in supine and tilt positions in the whole group of patients.

	Non-responders *N* = 14	Responders *N* = 60	*P*
Supine position			
Baseline values			
BREC	44.5 (16.0–55.0)	26.0 (14.0–45.5)	0.217
BRS	10.5 (8.9–13.2)	10.2 (6.5–13.8)	0.420
BEI	58.1 ± 15.5	51.2 ± 20.3	0.238
After ablation			
BREC	10.0 (2.0–24.0)	12.0 (4.0–21.5)	0.939
BRS	5.4 (3.7–6.5)	4.8 (3.6–7.2)	0.809
BEI	24.8 (15.9–27.4)	17.5 (8.9–27.5)	0.508
Tilt position			
Baseline values			
BREC	33.0 (13.0–45.0)	27.0 (9.0–44.5)	0.557
BRS	7.1 (5.5–8.6)	5.7 (4.9–8.8)	0.178
BEI	42.5 (38.3–48.4)	38.5 (23.8–53.0)	0.624
After ablation			
BREC	8.0 (1.0–19.0)	7.0 (3.0–17.5)	0.494
BRS	4.7 (3.8–6.2)	3.7 (2.8–5.2)	0.212
BEI	14.3 (5.0–26.1)	12.4 (4.9–22.0)	0.874

BREC, baroreceptor event count; BRS, baroreceptor slope mean; BEI, baroreceptor effectiveness index.

**Table 3:** The changes in baroreceptor parameters in non-responders and responders after ablation compared with baseline parameters in supine and tilt positions in the whole group.

	Non-responders *N* = 14	Responders *N* = 60	*P*
Supine position			
ΔBREC	−26.6 ± 25.5**	−15.2 ± 22.8***	0.103
ΔBRS	−5.0 (−8.1 to −3.6)***	−4.2 (−8.4 to −1.0)***	0.224
ΔBEI/BEI baseline	−37.4 ± 19.5***	−29.6 ± 23.5***	0.254
Tilt position			
ΔBREC/BREC baseline	−12.0 (−43.0 to −8.0)*	−11.0 (−33.0 to −2.0)***	0.498
ΔBRS/BRS baseline	−2.8 (−5.2 to −1.3)*	−2.1 (−4.4 to −0.8)***	0.591
ΔBEI/BEI baseline	−22.2 (−38.3 to −12.6)*	−24.5 (−35.7 to −9.3)***	0.715

BREC, baroreceptor event count; BRS, baroreceptor slope mean; BEI, baroreceptor effectiveness index.

* *p* < 0.05, **p < 0.01, ***p < 0.001 compared with baseline measurements.

**Table 4:** A comparison of BRS values in both ablation groups before and after AF ablation in supine and tilt positions.

	CB group			RF group			*P*	
	NR *N* = 5	R *N* = 34	*P*	NR *N* = 9	R *N* = 26	*P*	NR	R
Supine position								
Before ablation								
BREC	44.0 (16.0–55.0)	30.5 (21.0–42.0)	0.737	45.0 (22.0–53.0)	21.5 (9.0–46.0)	0.168	0.790	0.241
BRS	9.2 (9.0–10.2)	9.8 (6.3–13.8)	0.983	11.3 (8.9–13.4)	10.2 (7.1–13.2)	0.369	0.351	0.872
BEI	54.0 (50.0–70.6)	55.3 (41.6–73.2)	1.00	63.5 (47.2–66.7)	47.8 (32.6–60.0)	0.059	0.894	**0** **.** **037**
After ablation								
BREC	9.0 (6.0–18.0)	15.0 (10.0–22.0)	0.412	10.0 (2.0–24.0)	5.0 (2.0–16.0)	0.461	0.738	**0**.**047**
BRS	5.8 (5.6–5.8)	5.0 (3.8–7.0)	0.609	4.08 (3.2–6.5)	4.3 (3.2–8.2)	0.671	0.505	0.471
BEI	20.5 (15.9–21.8)	20.0 (11.8–28.9)	0.629	26.8 (23.8–27.4)	11.7 (7.7–24.1)	0.227	0.182	0.088
Tilt position								
Before ablation								
BREC	28.0 (27.0–45.0)	31.0 (10.0–42.0)	0.950	32.0 (13.0–43.0)	16.0 (5.0–45.0)	0.450	0.947	0.300
BRS	6.8 (5.5–7.1)	5.8 (4.7–9.2)	0.721	8.2 (6.5–8.6)	5.7 (5.0–8.0)	0.168	0.424	0.823
BEI	42.1 (41.1–44.7)	36.3 (25.7–56.5)	0.599	42.9 (38.3–48.4)	39.3 (22.2–52.1)	0.836	0.894	0.469
After ablation								
BREC	13.0 (1.0–19.0)	7.5 (3.0–20.0)	0.950	4.0 (0.0–18.0)	6.5 (4–15)	0.395	0.420	1.00
BRS	4.6 (4.6–4.7)	3.7 (3.3–5.0)	0.195	5.0 (1.2–7.2)	3.6 (2.8–5.3)	0.553	1.00	0.736
BEI	22.5 (7.5–25.1)	12.9 (4.2–22.1)	0.599	12.0 (5.0–17.0)	11.8 (6.1–17.6)	0.925	0.689	0.794

NR, non-responders; R, responders; CB, cryoballoon ablation; RF, radiofrequency ablation; BREC, baroreceptor event count; BRS, baroreceptor slope mean; BEI, baroreceptor effectiveness index.

Bold indicates values that are statistically significant.

**Table 5:** Changes in baroreceptor parameters in non-responders and responders after ablation compared with baseline parameters in supine and tilt positions in the CB and RF groups.

	CB group			RF group			*P*	
	NR (*N* = 5)	R (*N* = 34)	*P*	NR (*N* = 9)	R (*N* = 26)	*P*	NR	R
Supine position								
ΔBREC	−21.4 ± 26.0	−14.7 ± 25.1**	0.584	−29.6 ± 26.2*	−15.9 ± 19.9**	0.180	0.586	0.848
ΔBR	−3.6 (−4.4 to −3.5)	−4.6 (−8.3 to −0.8)***	1.00	−6.9 (−8.5 to −4.2)**	−3.9 (−9.3 to −1.7)***	0.180	0.110	0.461
ΔBEI	−39.3 ± 25.0*	−32.0 ± 24.2***	0.535	−36.3 ± 17.3***	−26.4 ± 22.6***	0.299	0.800	0.366
Tilt position								
ΔBREC	−12.2 ± 40.5	−19.7 ± 24.9***	0.564	−23.3 ± 20.8*	−15.5 ± 18.9***	0.304	0.527	0.473
ΔBRS	−2.2 (−2.4 to −0.7)]	−2.1 (−4.4 to −1.1)***	0.796	−4.7 (−6.6 to −1.9)	−2.3 (−4.3 to −0.3)***	0.316	0.256	0.888
ΔBEI	−28.5 ± 23.3	−26.3 ± 21.4***	0.817	−26.8 ± 19.5**	−23.5 ± 18.6***	0.657	0.881	0.600

NR, non-responders; R, responders; CB, cryoballoon ablation; RF, radiofrequency ablation; BREC, baroreceptor event count; BRS, baroreceptor slope mean; BEI, baroreceptor effectiveness index; CB, cryoballoon ablation; RF, radiofrequency ablation; BREC, baroreceptor event count; BRS, baroreceptor slope mean; BEI, baroreceptor effectiveness index.

* *p* < 0.05, **p < 0.01, ***p < 0.001 compared with baseline measurements.

During tilt provocation after ablation, the values of the BR parameters were significantly reduced, but they did not differ between non-responders and responders.

According to the AFSS, the global score of perceived AF burden was significantly decreased in both groups after 3, 6, and 12 months (Table 6). Patients in the CB group were characterized by a significantly lower score after 3 months in comparison with those in the RF group. Similarly, the global score was lower in the CB group than in the RF group after 6 and 12 months (Table 6). However, after adjustment for baseline values, these differences became non-significant (Table 7).

**Table 6:** A comparison of AF burden before and after ablation at 3, 6, and 12 months according to the AFSS.

AFSS	Cryoballoon ablation	Radiofrequency ablation	*P*
	Median [Q1–Q3]	Median [Q1–Q3]	
Baseline	6.0 [2.0–13.0]	10.5 [5.0–14.0]	0.199
3 months	2.5 [1.0–6.0]***	7.0 [1.0–12.0]	**0**.**046**
6 months	1.5 [0.0–4.0]***	6.0 [2.0–12.0]*	**0**.**012**
12 months	1.5 [0–4.0]**	6.5 [2.0–12.0]**	**0**.**029**

* *p* < 0.05, **p < 0.01, ***p < 0.001 compared with baseline measurements.

Bold indicates values that are statistically significant.

**Table 7:** A comparison of AF burden before and after ablation at 3, 6, and 12 months according to the AFSS adjusted for baseline values.

AFSS	Cryoballoon ablation	Radiofrequency ablation	*P*
	Median [Q1–Q3]	Median [Q1–Q3]	
3 months	3.7 [1.0–8.4]	8.7 [5.0–11.1]	0.131
6 months	2.4 [0.5–6.5]	7.2 [4.0–9.3]	0.064
12 months	2.5 [0.4–6.2]	7.1 [4.2–8.9]	0.064

## Discussion

Our study showed that the CA-induced decrease in the BR parameters, which is more pronounced in the CB group than in the RF group, is not associated with the 1-year CA efficacy.

Baroreceptors are a type of mechanoreceptors that secure a constant input in the overall regulation of BP homeostasis. Information about BP fluctuations is rapidly passed by the efferent part of the ANS, resulting in total peripheral resistance and cardiac output changes, maintaining BP within a preset, normalized range (15). Many investigations have been performed to assess the activity of the BR and its impact on BP homeostasis (16, 17), as BR dysfunction has been involved in multiple disease processes (18). The imbalance in BR sensitivity has been shown to play a role in the vasovagal syndrome (19), and the clinically significant reduction in BRS values is a predictor of cardiac mortality in patients with previous myocardial infarction (20).

It has been also shown that BRS values are depressed in patients with AF (21). An acute increase in the average heart rate following PVI, which suggests partial vagal denervation, has been demonstrated in several studies. This increase generally persists for up to 1 year and is associated with a decreased risk of AF recurrence (6–23).

In our study, we evaluated the activity of the ANS after the CA and attempted to assess the effectiveness of two different types of ablation based on BR values. It is commonly known that the efficacy of CB CA and RF CA is comparable (24, 25). Several studies have investigated the effects of PVI on the ANS, usually using HR and HRV parameters (7, 8). In the only study on the impact of cryoballoon ablation of AF on autonomic balance in a long-term observation, Oswald et al. (26) analyzed HR requiring stimulation during ablation and HRV from Holter before and 1 week, 1 month, and 3 months after ablation in 14 patients. The 6-month rate of effectiveness of the procedure was 64%. HRV decreased significantly immediately after ablation but gradually normalized by 3 months. Changes in HRV were similar in those treated both successfully and unsuccessfully. The studies assessing BR function following CA showed reduced BR values after CA (9, 21, 27, 28). Miyoshi et al. showed that catheter ablation depressed the BR function irrespective of the type of AF, with a greater effect on patients with paroxysmal than persistent AF (21), and Kondo et al. showed that a lack of decrease in the BR function after radio-frequency catheter ablation (RFCA) may be associated with procedural failure (27). It was also suggested that a depressed BR function may lead to a higher recurrence rate after CA in patients with persistent AF (21).

Recently, pulse field ablation (PFA) was implemented as a new method of AF ablation. It is generally considered that PFA is a method that spares nerves and adjacent tissues, and therefore, the effect of PFA on the parasympathetic nervous system can be expected to be less long-lasting than the effect of thermal ablation (RF or CB). However, the data are conflicting. Del Monte et al. showed that PFA is associated with only a transitory and short-lasting effect on the parasympathetic system (29). On the other hand, a subanalysis of an ADVENT study showed that PFA caused similar changes in HR increase as RF and CB (30). Because PFA is a relatively new method, there are still a number of issues that need investigation.

To date, the BR reflex has not been evaluated using tilt testing to compare the effectiveness of two different types of ablations in a long-term observation. CA contributes to the inhibition of the BR reflex by denervation of the GPs in the heart. The reduction of AF recurrences is achieved by denervating the GPs during PVI (6). The deeper penetration of freezing energy in the myocardial tissue during CB probably causes more damage to parasympathetic GPs than RF energy. However, the efficacy of CA was similar in both groups. This may suggest that, in some patients, the CA-induced changes in the autonomic control of the heart are not as important in achieving long-term success as in achieving complete PVI. It may be speculated that vagal denervation may be more important in selected patients with the so-called vagally mediated AF than in unselected consecutive patients undergoing CA, as in our study. This is in line with the discrepant results of other studies that assessed changes in the ANS following CA. In these studies, the effectiveness of PVI + GP ablation widely ranged between 50% and 91% (31, 32). When CB and RF were compared, there was no difference between the groups in terms of freedom from AT/AF or procedural complications (33).

Our study confirmed that CA of AF is associated with improved quality of life, both in the RF and in the CB groups. The AF symptoms were significantly reduced in both groups to a similar extent. Importantly, patients in the CB group were characterized by a significantly lower sum of points on the AFSS after 3 months of observations in comparison with those in the RF group. Similarly, the sum of points was lower at 6 and 12 months, respectively. This result is consistent with that of previous studies concerning the long-term effectiveness of CB and RF CA (22, 23).

### Strengths of the study

The strengths of the study are as follows: (1) A well-defined and homogeneous population of patients with paroxysmal AF. (2) Use of BRs to evaluate CA-induced changes in BR function. (3) Use of Holter monitoring as an objective way to assess AF recurrence. (4) Confirmation of BR attenuation following CA of AF. (5) Demonstrating that CB leads to a greater decrease in BR function than RF does, but the long-term effectiveness of both procedures is similar.

### Limitations

First, the number of patients included in the study was relatively small. The number of patients was too low to compare the predictive value of BR reflex changes on the outcome. Second, this study was an unblinded and non-randomized one; however, demographic and clinical characteristics were similar in both patient groups. Third, due to a lack of data, the effectiveness of CA was assessed based on only Holter monitoring and not compared with BRS values because of the fact that baroreflex function was assessed only shortly after ablation and not at a later time point. The fact that baroreflex function was assessed only shortly after ablation and not at a later time point did not allow an evaluation of longitudinal changes. Finally, we did not use other tools to assess BR parameters, such as extracardiac vagal stimulation (ECVS), but at the time of conducting the study, we had no access to the ECVS device.

## Conclusions

CA for AF significantly decreased the BR function, especially in patients undergoing CB. There was no correlation between CA-induced changes in BR parameters and ablation outcome.

## Data Availability

The raw data supporting the conclusions of this article will be made available by the authors without undue reservation.
